# Idiots

**Published:** 1857-07

**Authors:** 


### IDIOTS.

Idiotcy is arrested development. There is in all cases a deficiency of brain, a low physical organization, or functional disorganization.

The humane and accomplished Dr. Wilbur says, that out of a class of twenty pupils, only three could count ten. Their most universal fault was gluttony. Their great want is the power of attention. Many cannot talk; it often requires two or three years to enable them to utter a single word distinctly.

In almost all cases, home treatment only confirms the malady.

In three hundred and fifty-nine cases, all but four originated in parents who had brought on some confirmed disease by the violation of the laws of nature. In every single instance, the four excepted, either one or both parents were either very unhealthy, scrofulous, disposed to insanity, indulged in animal excesses, or had married blood relations. Let every reader commit to memory these five causes, for to have an idiotic child, how terrible the infliction!

More than one fourth of three hundred and fifty-nine idiots were the children of drunkards; one out of every twenty was the child of the marriage of near relations; in one such family five children out of eight were idiotic. If then, health, temperance, and chastity are not duties, then are we irresponsible.

